# Cinnamaldehyde Improves Lifespan and Healthspan in* Drosophila melanogaster* Models for Alzheimer's Disease

**DOI:** 10.1155/2018/3570830

**Published:** 2018-08-29

**Authors:** Hanh M. Pham, Anna Xu, Samuel E. Schriner, Evgueni A. Sevrioukov, Mahtab Jafari

**Affiliations:** Department of Pharmaceutical Sciences, University of California, Irvine, CA, USA

## Abstract

Cinnamon extract has been reported to have positive effects in fruit fly and mouse models for Alzheimer's disease (AD). However, cinnamon contains numerous potential active compounds that have not been individually evaluated. The main objective of this study was to evaluate the impact of cinnamaldehyde, a known putative active compound in cinnamon, on the lifespan and healthspan of* Drosophila melanogaster* models for Alzheimer's disease, which overexpress A*β*_42_ and MAPT (Tau). We found that cinnamaldehyde significantly improved the lifespan of both AD and non-AD flies. Cinnamaldehyde also improved the healthspan of AD flies overexpressing the Tau protein by improving climbing ability, evaluated by rapid iterative negative geotaxis (RING), and improving short-term memory, evaluated by a courtship conditioning assay. Cinnamaldehyde had no positive impact on the healthspan of AD flies overexpressing the A*β*_42_ protein.

## 1. Introduction

Alzheimer's disease (AD) results in neuronal dysfunction and locomotor impairment that would eventually lead to the loss of ability to carry out simple motor functions such as walking, talking, and holding objects [1, 2]. Other symptoms of AD include memory loss, confusion, and behavioral changes [1]. The exact etiology for AD is poorly understood, but it is generally accepted that the underlying pathology of AD is due to abnormal levels of beta-amyloid in the brain resulting in the accumulation of plaques between neurons and hyperphosphorylated Tau proteins resulting in the formation of tangles in neuronal cells [3]. Alleles of the apolipoprotein E (ApoE) gene and certain unhealthy lifestyle choices have also been proposed as possible contributors to AD by elevating A*β* and Tau in the brain [4]. For example, the ApoE4 allele has been associated with an increased risk of developing Alzheimer's disease, whereas ApoE3 has a neutral relationship and ApoE2 is negatively associated with the disease [5]. In the case of lifestyle changes, the occurrence of Alzheimer's disease has been linked to diet, social interaction, physical exercise, and mental activity [5].

*Drosophila melanogaster* models for AD that are based on overexpressing A*β* or Tau proteins have been used to evaluate not only the pathology of AD, but also the impact of potential interventions on the pathology and symptoms of AD [3, 6, 7]. These genetically modified models display similar neuronal dysfunction as seen in humans with AD such as neurodegeneration, neurotoxicity, and locomotion defects [3]. The overexpression of a Tau protein compromises the associative olfactory learning and memory as well as neurodegeneration of the fly [3]. The overexpression of A*β* results in the formation of diffuse A*β* deposits, gradual locomotor dysfunction, neurodegeneration, premature death, and learning defects [8]. Alzheimer's disease has also been associated with reduced gait speed, loss of muscle strength and bulk, and reduced balanced and dexterity [9]. However, these neurological manifestations are far less severe in AD than those found in either Huntington's or Parkinson's disease. While overexpression of different species of beta-amyloid, A*β*_40_ and A*β*_42_ can cause neuronal dysfunction and memory defects, only A*β*_42_ species cause neurodegeneration with amyloid deposits making the aggregation of A*β*_42_ and Tau proteins the pathological hallmark in AD brains [8]. Though it may be appropriate to use fly models overexpressing either A*β* or Tau protein to evaluate how compounds affect the AD pathology, a faithful model of AD should feature the overexpression of both A*β* and Tau, as accumulating evidence suggests that A*β*_42_ plays a central role in the pathogenesis of AD, and Tau acts downstream of A*β*_42_ as a modulator of the disease progression [10].

*Drosophila melanogaster* models for AD, AD flies, have also been used to investigate the impact of pharmaceuticals and natural products, such as cinnamon, on the pathology of AD. Cinnamon is widely used by humans, both as a spice and as a traditional medicine with reported therapeutic properties such as glucose lowering as well as antioxidant and antimicrobial effects [11]. The therapeutic benefits of cinnamon are often contributed to the activities of its putative active compounds such as cinnamaldehyde, eugenol, cinnamyl acetate, and cinnamyl alcohol [12]. With respect to AD, in a comprehensive review of the pharmaceutical and phytochemical applications of the cinnamon, the extract was reported to have an inhibitory effect on Tau aggregation in AD animal models with positive effects on lifespan and motility [13]. This review did not report which specific compounds in the extract were responsible for these biological activities. Since cinnamaldehyde is considered to be the most abundant compound in cinnamon, approximately 45-62% by weight [12], we hypothesized that cinnamaldehyde should have positive impacts on the lifespan and healthspan of AD flies.

It has been reported that 40 mM of cinnamaldehyde did not extend the lifespan of normal flies (non-AD flies) [11]. For this work, we tested a dosing range of 16 to 400 mM of cinnamaldehyde on fly lifespan to identify the optimal dose. Cinnamaldehyde lifespan studies were performed as described in Schriner et al. [11]. The effects of cinnamaldehyde on both the lifespan and healthspan of* Drosophila melanogaster* model for Alzheimer's disease were examined in this study. We evaluated healthspan phenotypes in this study by two validated tests: rapid iterative negative geotaxis (RING) and a courtship conditioning assay to examine short-term memory changes [11, 14, 15].

## 2. Materials and Methods

### 2.1. Cinnamaldehyde

Cinnamaldehyde was obtained from Sigma-Aldrich in liquid form. The certificate of analysis showed a purity of 99%. We performed a dose finding study and tested the impact of 16 to 400 mM of cinnamaldehyde on fly lifespan to identify the optimal dose. As described in Schriner et al. [11], cinnamaldehyde was fed to flies by adding it to the yeast paste that was placed on the top of standard banana food.

### 2.2. Fly Crossing

Parental control flies of APP.Abeta_42_, and MAPT (Tau), were crossed with the* Gal4*^*elav-C155*^* UAS* driver. This resulted in first-generation (F1) offspring expressing A*β*_42_ or MAPT, which served as our* Drosophila melanogaster* models for Alzheimer's disease (AD flies). Stock cages with 3% yeasted banana food plates were used to house the flies. After two days, the offspring eggs were collected and transferred to standard food to mature and hatch for 10 days. All flies in the assays in this work were maintained at 25°C and 55% humidity.

### 2.3. Survival Assay

As described by Schriner et al., the survival assay is a standard tool to evaluate the effect of genotype, interventions, or environmental conditions on the* Drosophila *lifespan [11]. This assay was performed to evaluate the lifespan of* Gal4*^*elav-C155*^ lines that express toxic proteins in their central nervous system. The number of dead flies among cohort produced by the* Gal4*^*elav-C155*^ crossings was recorded every other day. For this assay, 240 male and 240 female flies were transferred to fresh vials with standard food every two days until all flies had died. A total of 12 flies, 6 males and 6 females, were housed per vial.

### 2.4. Rapid Iterative Negative Geotaxis (RING) Assay

As described by Schriner et al., the RING assay was conducted to evaluate the impact of cinnamaldehyde on fly directionality and climbing ability [11]. This assay was used to measure locomotor ability. A total of 6 trials were tested for each group. In each trial, 20 flies supplemented with either cinnamaldehyde or control diet were placed in an empty vial and covered with a sponge. The camera was positioned to view and record the vials vertically. Flies were then tapped down to the bottom of their vials. A snapshot of the location of flies after 4 seconds was taken and the average travelled distance of 20 flies was calculated.

### 2.5. Courtship Conditioning

As described by Koemans et al., courtship conditioning was performed in 2 separate periods: a training period where learning occurs and a testing period to evaluate memory ability [15]. Five- to seven-day-old wild-type Oregon-R strain females, used for training and testing, were premated before the training period. To prepare premated female flies, a virgin Oregon-R female was paired with an Oregon-R male for 24 hours. To train the virgin male AD flies, flies were introduced to premated Oregon-R female flies for one hour and were allowed to initiate and learn a courtship condition that paired mating rejection with the pheromone excreted by the premated female as described in Koemans et al. [15]. One hour after the training period, the trained male flies were separated into new vials and left undisturbed to rest for one hour. The one-hour resting period was used to evaluate memory performance and memory retention from the training as well as increasing their sensitivity [15]. After this one-hour resting period, each trained male was paired with a new premated Oregon-R female fly in a new food vial. The testing period was initiated and the flies were observed and recorded for 10 minutes. The courtship behavior time of individual male flies was recorded and the courtship index (CI) was determined by dividing the total time of male courtship behavior by 10 minutes. The CI was calculated for both naïve and trained male flies [15]. The memory index (MI) was then determined by subtracting the courtship index (CI) of trained male flies from the CI of naïve male flies, then dividing this by the CI of the naïve flies [15]. All male AD (APP.Abeta_42,_ MAPT (Tau)) flies were maintained at 25°C and transferred to vials with fresh food every other day for 20 days before the assays were conducted [15]. Treated flies were fed 80 mM of cinnamaldehyde for 20 days after eclosion. Training and testing were performed on 20-day-old male flies.

### 2.6. Statistical Analysis

Data analysis was done using GraphPad Prism. Student's* t*-test was performed when comparing between means of two groups. A* P* value of <0.05 was considered significant.

## 3. Results

### 3.1. Cinnamaldehyde Increased the Lifespan of AD, Non-AD, and Parental Control Flies

**Figure 1:**
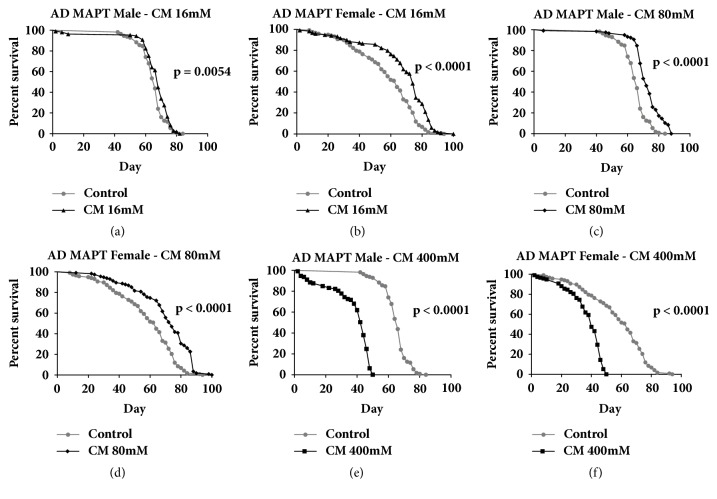
**The effect of cinnamaldehyde on the lifespan of Alzheimer's disease (AD) flies overexpressing MAPT (Tau). **(a, b) 16 mM cinnamaldehyde (CM) significantly increased the lifespan of AD flies overexpressing the Tau protein in males by 2% and females by 17.5% (*P*=0.0054, male control n = 112, male CM n = 109,* P* < 0.0001, female control n = 118, and female CM n = 118). (c, d) 80 mM cinnamaldehyde significantly increased the lifespan of AD flies overexpressing the Tau protein in males by 11.7% and in females by 20.7% (male control n = 112, male CM n = 117, female control n = 118, and female CM n = 115,* P* < 0.0001). (e, f) 400 mM cinnamaldehyde was toxic to AD flies overexpressing the Tau protein in both males and females (male control n = 112, male CM n = 113, female control n = 118, and female CM n = 118,* P* < 0.0001).

**Figure 2:**
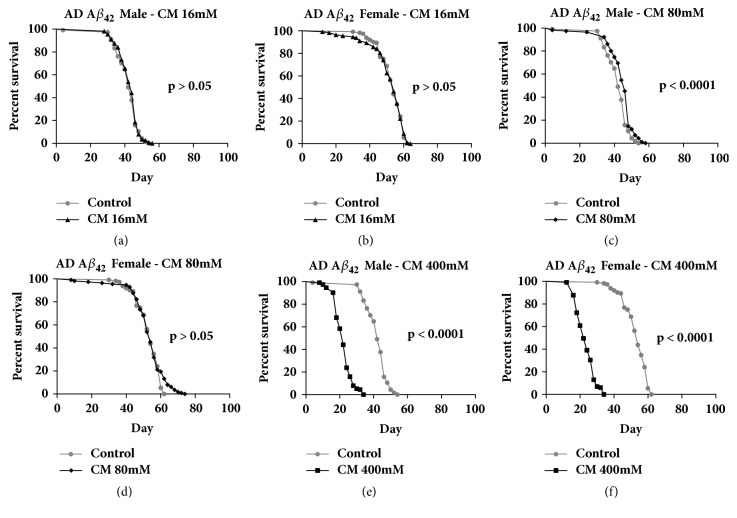
**The effect of cinnamaldehyde on the lifespan of Alzheimer's disease (AD) flies overexpressing A*β***_**42**_. (a, b) 16 mM of cinnamaldehyde (CM) had no significant effect on the lifespan of AD flies overexpressing the A*β*_42_ protein (male control n = 114, male CM n = 113, female control n = 112, and female CM n = 112,* P* > 0.05). (c, d) 80 mM of cinnamaldehyde significantly increased the lifespan of male AD fly model overexpressing the A*β*_42_ protein by 5.7% (male control n = 114, male CM n = 115,* P* < 0.0001) and it had no significant impact on the lifespan of females (female control n = 112, female CM n = 113,* P* > 0.05). (e, f) 400 mM of cinnamaldehyde was toxic to AD flies overexpressing the A*β*_42_ protein in both males and females (male control n = 114, male CM n = 113, female control n = 112, and female CM n = 115,* P* < 0.001).

**Figure 3:**
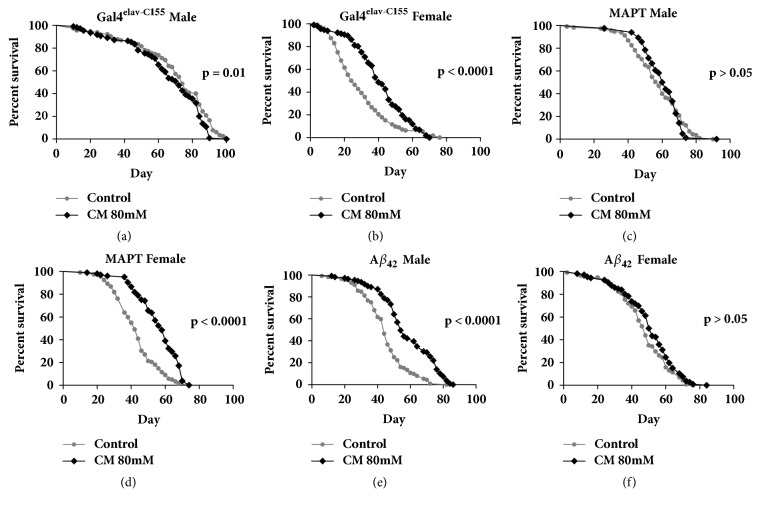
**The effect of 80 mM of cinnamaldehyde on the lifespan of non-Alzheimer's disease (AD) parental control flies. **(a, b) Cinnamaldehyde significantly increased the lifespan of male Gal4^elav-C155^ parental control flies by 5.4% (male control n = 115, male CM n = 110,* P* = 0.01) and females by 39.9% (female control n = 109, female CM n = 111,* P* < 0.0001). (c) Cinnamaldehyde had no effect on the lifespan of non-AD MAPT parental control males (male control n = 113, male CM n = 84,* P* > 0.05). (d) Cinnamaldehyde increased the lifespan of non-AD MAPT parental control females by 30.9% (female control n = 121, female CM n = 104,* P* < 0.0001). (e) Cinnamaldehyde increased the lifespan of non-AD A*β*_42_ parental control males (male control n = 110, male CM n = 109,* P* < 0.0001). (f) Cinnamaldehyde had no effect on the lifespan of non-AD MAPT parental control females (female control n = 110, female CM n = 104,* P* > 0.05).

The impact of three different cinnamaldehyde doses (16 mM, 80 mM, and 400 mM) on lifespan was evaluated. Cinnamaldehyde at 16 mM had no significant effect on the lifespan of AD flies overexpressing A*β*_42_ but at 400 mM, it had toxic effects on the lifespans of both male and female AD flies expressing A*β*_42_. Cinnamaldehyde at 80 mM increased the lifespan of Alzheimer's disease fly models that overexpress the Tau protein by 11% in males (*P* < 0.0001) and by 20.7% in females (*P* < 0.0001) (Figure 1). Cinnamaldehyde at 80 mM increased the lifespan of Alzheimer's disease fly models that overexpress A*β*_42_ protein by 5.3% in males only (*P* < 0.0001) (Figure 2). In addition, cinnamaldehyde significantly increased the lifespan of conventional Gal4^elav-C155^ flies for both sexes and the non-AD parental control flies that had no abnormal expression of the male A*β*_42_ and female Tau proteins (Figure 3). There was no significant improvement on the lifespan of the parental control male Tau and female A*β*_42_. The differences in the male and female fly's physiology and the biological engineered gene could explain these sex-specific observations where they could affect antiaging mechanism differently in the male and female of each transgenic fly strain.

### 3.2. Cinnamaldehyde Improved Locomotion

**Figure 4:**
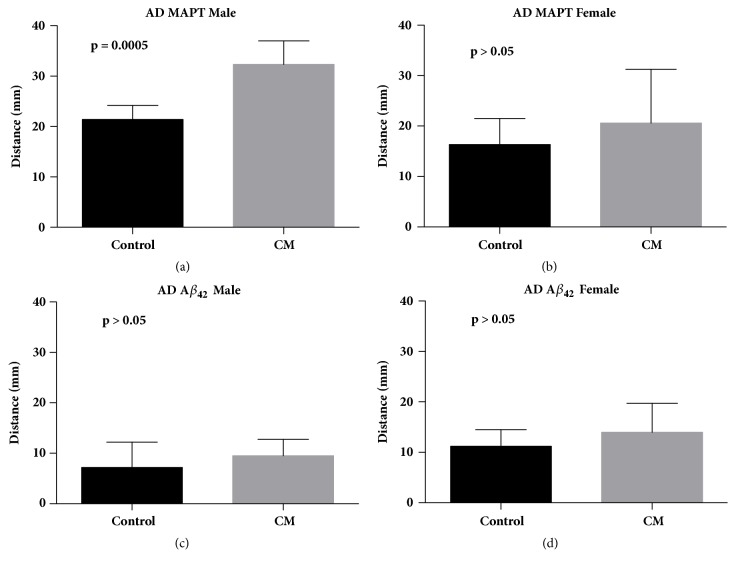
**The effect of 80 mM of cinnamaldehyde (CM) on climbing ability of 20-day-old* Drosophila melanogaster *model for Alzheimer's disease overexpressing MAPT (Tau) and A*β***_42_ (a) Cinnamaldehyde significantly increased climbing ability of 20-day-old male Alzheimer's disease flies overexpressing Tau (*P* = 0.0005, control n = 123, CM n = 120). (b) Cinnamaldehyde showed no significant impact on climbing ability of 20-day-old female Alzheimer disease flies expressing Tau (*P* > 0.05, control n = 112, CM n = 115). (c) Cinnamaldehyde showed no significant impact on climbing ability of treated 20-day-old male Alzheimer disease flies expressing A*β*_42_ (*P* > 0.05, control n = 109, CM n = 121). (d) Cinnamaldehyde showed no significant impact on climbing ability of treated 20-day-old female Alzheimer disease flies expressing A*β*_42_ (*P* > 0.05 control n= 112, CM n = 121).

**Figure 5:**
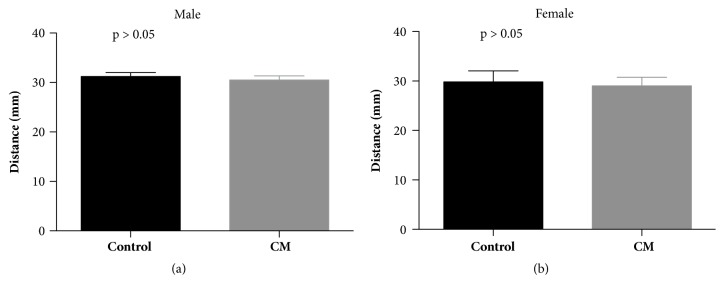
**The effect of 80 mM of cinnamaldehyde (CM) on climbing ability of 20-day-old wild-type Oregon-R (OR-R)* Drosophila melanogaster.***  No significant difference was observed in the climbing ability of control flies and flies treated with cinnamaldehyde in both male and female flies (male control n = 116, male CM n = 124, female control n = 116, and female CM n = 124).

The healthspan of AD flies was examined through the rapid iterative negative geotaxis (RING) assay that evaluates locomotion. Cinnamaldehyde significantly improved the climbing ability of male AD flies overexpressing Tau protein (Figure 4). This effect was specific to AD flies, as there was no significant improvement in climbing ability of wild-type (Oregon-R) flies treated with cinnamaldehyde (Figure 5).

### 3.3. Cinnamaldehyde Improved Short-Term Memory

**Figure 6:**
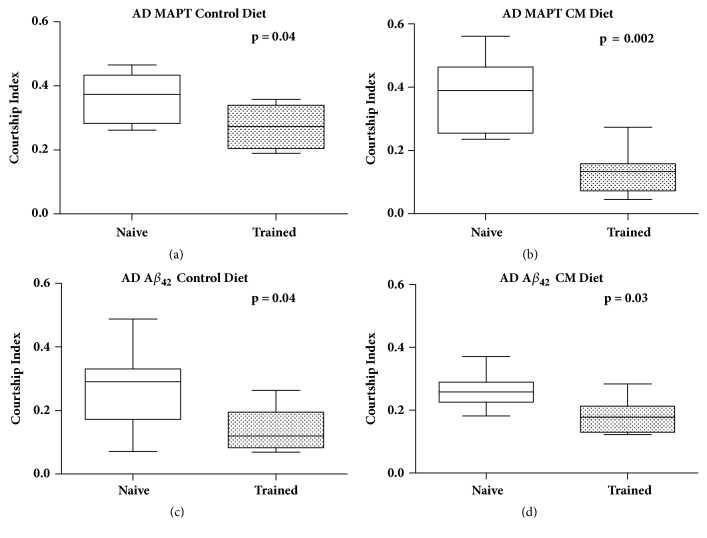
**The effect of training on courtship behavior in AD flies overexpressing either MAPT or A*β***_42_. The courtship index was significantly decreased after training in flies fed control diet ((a) and (c)), and 80 mM cinnamaldehyde ((b) and (d)) of both fly models for AD with overexpression of MAPT or A*β*_42_. ((a).* P* = 0.04, naïve n = 46, trained n = 44; (b).* P* = 0.002; naïve n = 40, trained n = 48; (c).* P* = 0.04; naïve n = 42, trained n = 46; (d).* P* = 0.03; naïve n = 45, trained n = 44)_._

**Figure 7:**
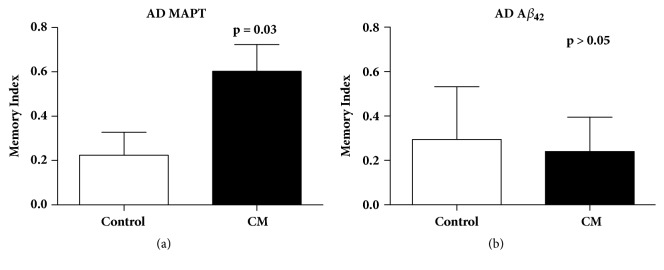
**The effect of 80 mM of cinnamaldehyde on short-term memory of AD flies overexpressing the MAPT (Tau) protein. **(a) Cinnamaldehyde significantly improved the memory index of AD flies overexpressing the Tau protein (*P*=0.03). (b) Cinnamaldehyde had no significant improvement in memory index of AD flies overexpressing A*β*_42_ protein (*P *> 0.05).

The effect of cinnamaldehyde on short-term memory through courtship and mating in the* Drosophila melanogaster *models for AD was also examined using a validated conditioned courtship assay as described in Koemans et al. [15]. The courtship index was first examined to determine whether training was conducted successfully. Here, the courtship index was significantly decreased after training and flies were fed either control diet or 80 mM cinnamaldehyde in both fly models of AD (Figure 6)_. _The observed decrease in courtship index demonstrated successful training as the trained male flies spent less time performing courtship with the new premated female than the naïve (untrained) male flies. We also observed that cinnamaldehyde supplementation led to a significant improvement in short-term memory for the AD flies overexpressing the Tau protein, but not AD flies overexpressing A*β*_42_ (Figure 7).

## 4. Discussion

Cinnamon extract has been reported to improve the lifespan and healthspan of* Drosophila melanogaster *models for Alzheimer's disease (AD) [13]. In this study, we evaluated the impact of cinnamaldehyde, the predominant putative active compound in cinnamon, on the lifespan and healthspan of* Drosophila melanogaster* models for AD. While the exact etiology of later-life AD is still far from clear, the pathology of AD is postulated to be due to the accumulation of extracellular plaques between the neurons from abnormal levels of beta-amyloid and the formation of neurofibrillary tangles from the hyperphosphorylation of Tau proteins. Thus, the majority of interventional studies in AD focus on the inhibition of the aggregation of A*β* and the tangles of the Tau protein.

We had previously reported that cinnamon increased lifespan in both males and females of two different strains of flies,* w*^1118^ and* JIV*, and that two putative active compounds in cinnamon, cinnamaldehyde (40 mM) and coumarin (35 mM), not only failed to increase the lifespan of these flies but they also appeared to have sex-specific toxicity [11]. We were not able to draw any conclusions on the impact of lower doses of these compounds on lifespan since we did not perform a dose finding assay. Since cinnamaldehyde is considered the most abundant compound in cinnamon, approximately 45-62% [12], we hypothesized that the lifespan extension that we observed with cinnamon could be due to cinnamaldehyde and that this compound might have positive impacts on healthspan for AD fly models. In order to determine the optimal dose of cinnamaldehyde that could effectively rescue the fly models that express MAPT (Tau) and A*β*_42_ a dose finding assay was performed and the impact of three different cinnamaldehyde doses (16 mM, 80 mM, and 400 mM) on lifespan was evaluated. Since cinnamaldehyde at 80 mM significantly increased the lifespan of both MAPT (Tau) and A*β*_42_ male flies by 11.7% and 5.7%, respectively (p<0.05), this dose was selected as the optimal dose for other assays such as locomotion and short-term memory. We also observed that while this dose significantly increased the lifespan of female AD flies that overexpressed Tau proteins by 20.7%, there was no significant impact of that dose on the lifespan of female AD flies that overexpressed A*β*_42_ proteins. We also evaluated the impact of 80 mM of cinnamaldehyde on the lifespan of three groups of non-Alzheimer disease parental control flies (non-AD A*β*_42_, Gal4^elav-C155^, and non-AD MAPT). Cinnamaldehyde significantly increased the lifespan of these three groups, none of which had abnormal expression of A*β*_42_ and/or Tau proteins. Since cinnamaldehyde increased the lifespan of AD and non-AD flies, independent of abnormal expression of A*β*_42_ and/or Tau proteins, it appears that the compound acts to extend lifespan through a pathway that is not related to Alzheimer's disease pathology.

We also evaluated the impact of cinnamaldehyde on the climbing ability of AD flies using the rapid iterative negative geotaxis (RING) assay. Cinnamaldehyde significantly increased the climbing ability of male AD flies overexpressing the Tau protein, but it did not improve the climbing ability of wild-type (Oregon-R) flies. The compound also improved short-term memory of male AD flies overexpressing the Tau protein. Since the positive impact of cinnamaldehyde was only observed in AD flies overexpressing the Tau protein, the positive effects of this compound could be due to disrupting Tau aggregation. In a study that evaluated the impact of epigallocatechin-3-gallate (EGCG) from green tea on the clearance of pathological Tau species in a cultured cell model, EGCG enhanced the clearance of phosphorylated Tau species in primary neurons [16]. Since phosphorylated Tau isoforms form the majority of Tau species in AD pathogenesis, their clearance could potentially be therapeutic [16].

The mechanism through which cinnamaldehyde improves the phenotype of the AD MAPT flies is speculative at this point. One possible explanation is that cinnamaldehyde works in a similar manner to that shown for the natural product epigallocatechin-3-gallate (EGCG), by directly disrupting Tau aggregation. Direct binding of EGCG to Tau has been reported, along with alteration of the 3D structure of Tau [17]. The effectiveness of EGCG has also been demonstrated by its ability to enhance Tau clearance in neuronal cultures and its ability to decrease the levels of soluble phosphorylated Tau in transgenic mice [16, 18].

Alternatively, cinnamaldehyde could be working in a similar manner to that of another natural product, sulforaphane, which enhances Tau degradation through the activation of nuclear factor erythroid 2-related factor 2 (Nrf2) [16, 19]. Oxidative stress, which activates Nrf2, is thought to be one of the underlying factors driving AD. Once activated, Nrf2 binds antioxidant response elements (ARE) to induce gene expression of downstream protective enzymes. One of these proteins is the autophagy adaptor protein NDP52, which, when expressed, can induce autophagy and clear Tau aggregates from the neurons [19].

In this study, the positive phenotypic impacts of cinnamaldehyde were only observed in AD flies overexpressing the Tau protein. Future experiments will be needed to precisely determine the mechanism through which cinnamaldehyde may impact Tau aggregation in fly models for AD.

## 5. Conclusion

The etiology of the complex neurodegenerative Alzheimer's disease is still far from being clear. It is speculated that the pathology of AD is due to abnormal levels of beta-amyloid in the brain that causes the accumulation of plaques between neurons and hyperphosphorylation of the Tau proteins that causes the formation of tangles within neuronal cells. It has been reported that cinnamon has an inhibitory effect on Tau aggregation of mouse and fruit fly models for AD and it has also been reported to have positive effects on lifespan and locomotion of these model species [13]. In this work, we found that cinnamaldehyde, the most abundant putative active compound of cinnamon, significantly improved lifespan and healthspan of male AD flies overexpressing the Tau proteins. Although the compound had no effect on flies overexpressing the A*β*_42_ proteins, due to its safety profile, it may still be considered as a potential therapy to alleviate a known underlying cause of AD pathology. Further studies in mammalian model systems are needed to evaluate the safety and efficacy of cinnamaldehyde as a potential therapy for AD.

## Data Availability

The data used to support the findings of this study are available from the corresponding author upon request.
